# Increased Expression of Cathepsin L: A Novel Independent Prognostic Marker of Worse Outcome in Hepatocellular Carcinoma Patients

**DOI:** 10.1371/journal.pone.0112136

**Published:** 2014-11-10

**Authors:** Jian Ruan, Hang Zheng, Wenguang Fu, Peng Zhao, Ning Su, Rongcheng Luo

**Affiliations:** 1 Cancer Center, Traditional Chinese Medicine-Integrated Hospital, Southern medical University, Guangzhou, Guangdong, People’s Republic of China; 2 Department of Hepatobiliary Surgery, The Affiliated Hospital of Luzhou Medical College, Luzhou, Sichuan, People’s Republic of China; 3 Department of Medical Oncology, The First Affiliated Hospital, School of Medicine, Zhejiang University, Hangzhou, Zhejiang, People’s Republic of China; 4 Department of Oncology, Guangzhou Chest Hospital, Guangzhou, Guangdong, People’s Republic of China; University of North Carolina School of Medicine, United States of America

## Abstract

**Objectives:**

To investigate the expression and role of Cathepsin L (CTSL) in Hepatocellular carcinoma (HCC) tissue and cell line (MHCC-97H), and to evaluate the clinical and prognostic significance of CTSL protein in patients with HCC.

**Methods:**

The expression of CTSL was examined in HCC tissue and MHCC-97H cells by Western-blotting, Real-time PCR and immunohistochemical staining. Cell growth curve assay and colony formation assay were used to verify the effect of CTSL on the proliferation and tumor progression ability of MHCC-97H cells. Tumor formation assay in nude mice was used to analyze the effect of CTSL on the tumorigenicity of MHCC-97H cells.

**Results:**

The status of CTSL protein in carcinoma tissues is much higher than that in paracarcinoma tissues. The overall survival of the patients with high CTSL expression was significantly shorter than the low CTSL expression group. high CTSL expression was significantly correlated with advanced clinical staging, histological grade and tumor recurrence. In vitro experiments demonstrated that over-expression of CTSL in MHCC-97H cells promoted cell proliferation and tumor progression ability. Down-regulation of CTSL showed the opposite effects. Over-expression of CTSL increase the tumorigenicity of MHCC-97H cells by in vivo experiments. Moreover, multivariate analysis suggested that CTSL expression might be an independent prognostic indicator for the survival of HCC patients after curative surgery.

**Conclusions:**

CTSL might involve in the development and progression of HCC as a oncogene, and thereby may be a valuable prognostic marker for HCC patients.

## Introduction

Hepatocellular carcinoma (HCC) is a highly lethal cancer whose prognosis is poor. It ranks the third cause for cancer deaths in East Asia and sub-Saharan Africa, and the second for male cancer deaths in China [1]. Now, the incidence of HCC is also increasing in the United States and Europe [2]. Surgical resection remains to be the standard choice of treatment for patients in the early stage of HCC. However, even with radical resection, 60–70% of patients develop metastasis and recurrence within five years after surgery. Although several clinicopathological features including a poorly differentiated phenotype, large-sized tumor, and portal venous invasion have been found to contribute to the poor prognosis in HCC patients before operation, the underlying molecular mechanisms of the development of HCC remain unclear. Thus, it is urgent to study the pathogenesis of HCC.

CTSL, a lysosomal endopeptidase expressed in most eukaryotic cells, is a member of the papain-like family of cysteine proteinases [3]. Although commonly recognized as a lysosomal protease, CTSL is also secreted. This broad-spectrum protease is potent in degrading several extracellular proteins (laminins, fibronectin, collagens I and IV, elastin, and other structural proteins of basement membranes) as well as serum proteins and cytoplasmic and nuclear proteins [4]. CTSL plays a major role in antigen processing, tumor invasion and metastasis, bone resorption, and turnover of intracellular and secreted proteins involved in growth regulation [5], [6], [7], [8], [9], [10], [11], [12]. Increased CTSL level was found in multiple tumor types and associated with short survival of several cancers [13], [14], [15], [16], [17], [18], [19].

However, no research on CTSL has been done in HCC so far. To explore the exact role of CTSL in HCC, we investigated whether the expression of CTSL protein is different between tumor tissues and normal tissues, whether CTSL has any role in the development and progression of HCC, and whether CTSL is a prognostic factor in HCC after curative surgical treatment.

## Materials and Methods

### Patients and Specimens

Fresh tumor tissue samples with paired non-cancerous liver tissue samples of 26 HCC patients were obtained in operation from the Nanfang hospital. A total of 82 paraffin-embedded HCC samples, which were histologically and clinically diagnosed in patients with radical surgery in Nan Fang hospital, between 2000 and 2003, were also included in this study. Resected specimens, fixed in 10% formalin solution and then embedded in paraffin, were longitudinally sliced into 4-mm-thick sections. Representative sections were prepared and stained with hematoxylin and eosin for histologic examination. Western-blot was used to confirm the specificity of CTSL staining in fresh HCC tissues with paired non-cancerous liver tissues and MHCC-97H cell line. None of these patients had received radiotherapy or chemotherapy prior to surgical treatment. Clinical and pathological data of the 82 patients with HCC were collected, such as age, tumor size, stage, differentiation grade and recurrence. The tumor stages were classified according to the 2002 TNM staging system of Union for International Cancer Control (UICC). Tumor differentiation was classified using the Edmondson grading system. Clinical follow-up information was obtained by telephone or from the outpatient records.

Written Ethics Approval and Patient Consent from the Nanfang Hospital Research Ethics Committee were obtained. Participants were recruited and human experimentation was conducted in Nanfang Hospital. We have obtained written informed consent from all participants involved in the study.

### Cell Culture

The HCC cell lines MHCC-97H, MHCC-97L, Huh-7, HepG2, SMMC-7721, Bel-7404 and human colorectal carcinoma cell lines (CaCO2 and LoVo) were obtained from The Cell Bank of Type Culture Collection of Chinese Academy of Sciences. MHCC-97H cells were established from the cell line MHCC97. Spontaneous pulmonary metastasis occurred in 100% of recipient nude mice after orthotopic inoculation of MHCC-97H. Cells were maintained in RPMI 1640 medium (Gibco, Invitrogen, Carlsbad, CA) supplemented with 10% fetal bovine serum (FBS; Hyclone, Logan, UT), penicillin (100 units/ml), and streptomycin (100 units/ml) at 37°C in humidified 5% CO_2_ incubator.

### Western Blotting Analysis

Cell and tissue samples were solubilized in SDS lysis buffer, and the protein concentrations were detected by the BCA protein assay kit (PIERCE, Rockford, IL). Equal amounts of protein samples (30 µg/lane) were separated by electrophoresis through 9.0% resolving SDS–polyacrylamide gel, and then transferred to PVDF membranes (Amersham Pharmacia Biotech Inc in Piscataway, NJ). Block the non-specific binding sites by immersing the membrane into 5% non-fat milk in TBST solution for 1 hr, and then incubate the membrane with a primary monoclonal antibody to CTSL (Santa Cruz Biotechnology) for 2 hr at room temperature (RT). After washing 3 times in with TBST (TBS+0.5% Tween-20), the membranes were incubated with a secondary antibody (diluted 1∶1000 in TBS-T) for 1 hr at RT. The membranes were washed and proteins were detected by enhanced chemiluminescence system (Amersham Pharmacia Biotech) following manufacturer’s instructions. Anti-GAPDH mouse monoclonal antibody was used to confirm equal loading of lysates (1∶1000; Santa Cruz Biotechnology). Image J software was used to analyze the gray value.

### Real-time RT-PCR Analysis

Total RNA from human tissues was extracted using Trizol reagent (Invitrogen) according to the manufacturer’s instructions. cDNA was synthesized from 1 µg of total RNA by use of the SuperScript III First-Strand Synthesis System (Invitrogen). Real-time PCR was carried out using CFX96 Real-Time System (BIO-RAD). SYBR green 2× master mixture (Invitrogen) was used in a total volume of 10 µL. The primer sequences were as follows: CTSL sense 5′- CTGGTGGTTGGCTACGGATT-3′, antisense 5′-CTCCGGTCTTTGGCCATCTT-3′, GAPDH sense 5′-TGTTGCCATCAATGACCCCTT-3′, antisense 5′-CTCCACGACGTACTCAGCG-3′, GAPDH was used as an internal control. All reactions were run in triplicate in three independent experiments.

### Immunohistochemical Analysis

Immunohistochemical (IHC) staining was performed using Dako Envision system (Dako, Carpinteria, CA) according to the manufacturer’s instructions. The paraffin-embedded specimens were cut into 4 mm sections and baked 1 h at 65°C. All sections were deparaffined with xylenes and rehydrated through graded ethanol series to distilled water. Then, the sections were submerged into EDTA antigenic retrieval buffer (pH 8.0) and microwaved for antigenic retrieval. The sections were treated with 0.3% H_2_O_2_ for 15 min to block the endogenous peroxidase at RT, and then were treated with normal goat serum for 30 min to reduce the nonspecific binding. Consequently, the sections were incubated with rabbit polyclonal anti-CTSL antibody (1∶50; Santa Cruz Biotechnology) overnight at 4°C. After washing, the sections were incubated with biotinylated anti-goat secondary antibody (Zymed) followed by further incubation with streptavidin-horse-radish peroxidase (Zymed) at 37°C for 30 min. Diaminobenzidine (DAB) was used for color reaction, and the antibody was replaced by normal goat serum for negative controls.

The immunohistochemically stained tissue sections were scored independently by two pathologists blinded to the clinical parameters, and the final score was the average of the scores by two observers. We used the intensity and extent of the staining to evaluate the expression of CTSL. The staining intensity was scored as 0 (no staining), 1 (weak staining exhibited as light yellow), 2 (moderate staining exhibited as yellow brown), 3 (strong staining exhibited as brown). Extent of staining was scored as 0 (0%), 1 (1to 25%), 2 (26 to 50%), 3 (51 to 75%), and 4 (76 to 100%), according to the percentages of the positive staining areas relative to the whole carcinoma area or entire section for the normal samples. The sum of intensity and extent score was used as the final staining scores (0 to 7) for CTSL. For the purpose of statistical evaluation, tumors having a final staining score of <3 classified tumors with low CTSL expression and score >3 classified as high CTSL expression.

### Vector construction and transfection

The pcDNA3.0 vector was used to generate pcDNA-CTSL. The CTSL shRNA Plasmid was purchased from Santa Cruz Biotechnology (Cat. No: sc-29939-SH). Vector transfection was performed according to the instructions, MHCC97H cells and CaCO2 cells were transfected with pcDNA expressing CTSL or empty vector and MHCC97H were also used to knock-down the expression of CTSL. MHCC97H cells and CaCO2 cells expressing CTSL or empty vector were selected for 14 days with G418 after infection. MHCC97H transfected with CTSL-shRNA was selected for 14 days with puromycin after infection.

### Colony Formation Assay

For colony formation assay, cells were seeded evenly in 6-well plates (2×10^2^ cells per well) and cultured for 14 days. Then the cells were fixed with methanol for 10 min, stained with 1% crystal violet for 1 min. Each group of cells was performed in triplicate.

### 3-(4, 5-dimethylthiazol-2-yl)-2, 5-diphenyltetrazolium Bromide Reduction (MTT) Assay

Cells were seeded into 96-well plates at 2000 cells/well. Each sample had four replicates. The cells were incubated with 0.2% MTT for 4 h at 37°C, 100 µl DMSO/well was added to the culture cells to dissolve the crystals, and cells were counted every day by reading the absorbance at 490 nm.

### Tumor Formation in an Animal Model

Equivalent amounts of MHCC97H-CTSL cells and MHCC97H-Con cells (5×10^5^ cells) were injected subcutaneously into the right flank of female BALB/c nude mice (Shanghai Slac Laboratory Animal Co. Ltd, Shanghai, China) at 5 weeks of age (15–17.5 g). Tumorigenesis procedure was observed by measuring solid tumors in 3 dimensions with a caliper for 21 days. Animals were sacrificed 21 days after injection. The experiments on mice had been approved by the ethics committee at Nanfang hospital.

### Statistical Analysis

Statistical analyses were performed using a statistical software package (SPSS13.0, Chicago, IL). The significance of CTSL mRNA levels was determined by t-test. The chi-square test was used to analyze the relationship between CTSL expression and clinicopathological characteristics. Survival times were evaluated using the Kaplan and Meier survival curves, and compared by the log-rank test. The significance of various variables for survival was analyzed by multivariate survival analysis using Cox’s regression model. P-value less than or equal to 5 percent were considered to be statistically significant.

## Results

### The Expression of CTSL in HCC Tissues

**Figure 1:**
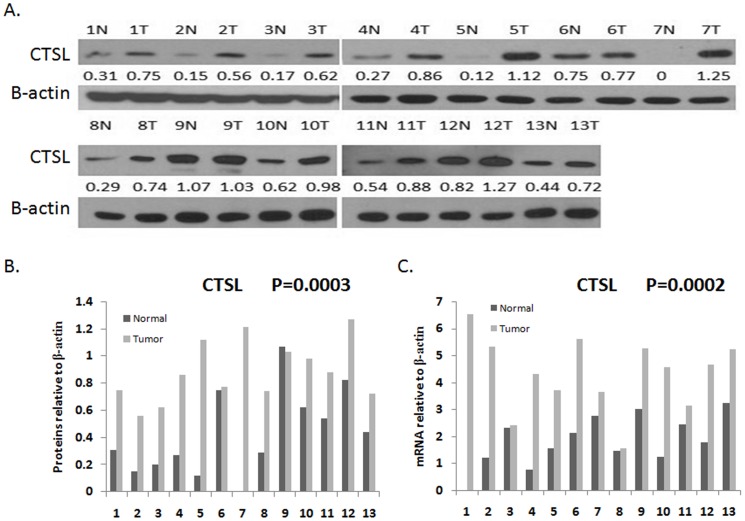
Expression levels of CTSL in HCC tissues. A. Expression levels of CTSL protein in 13 paired HCC tissues by Western blotting. N, paracarcinoma (normal) liver tissues. T, HCC tissues. B. Quantitative analysis of CTSL protein in 13 paired HCC tissues. C. mRNA levels of CTSL in 13 paired HCC tissues by real-time PCR.

To determine the expression of CTSL protein in HCC tissues, Western blotting was performed in 13 HCC tissues with paired non-cancerous tissues. Among 11 of 13 HCC tissues with paired normal tissues, clearly increased levels of CTSL expression was detected in all the tumors tissues in comparison to the paired non-cancerous tissues (Figure 1A and 1B). However, the levels of CTSL expression were similar in both tumors tissues and non-cancerous tissues in the rest 2 paired HCC tissues (Figure 1A, patient samples No. 6 and No. 9). We then determined whether the increased expression of CTSL occurred at mRNA level. We obtained an extra 13 paired HCC samples for real-time RT-PCR analysis. As shown in Figure 1C, the expression level of CTSL mRNA is significantly higher in tumor tissues. These data suggested that CTSL might serve as a oncogene in HCC.

**Figure 2:**
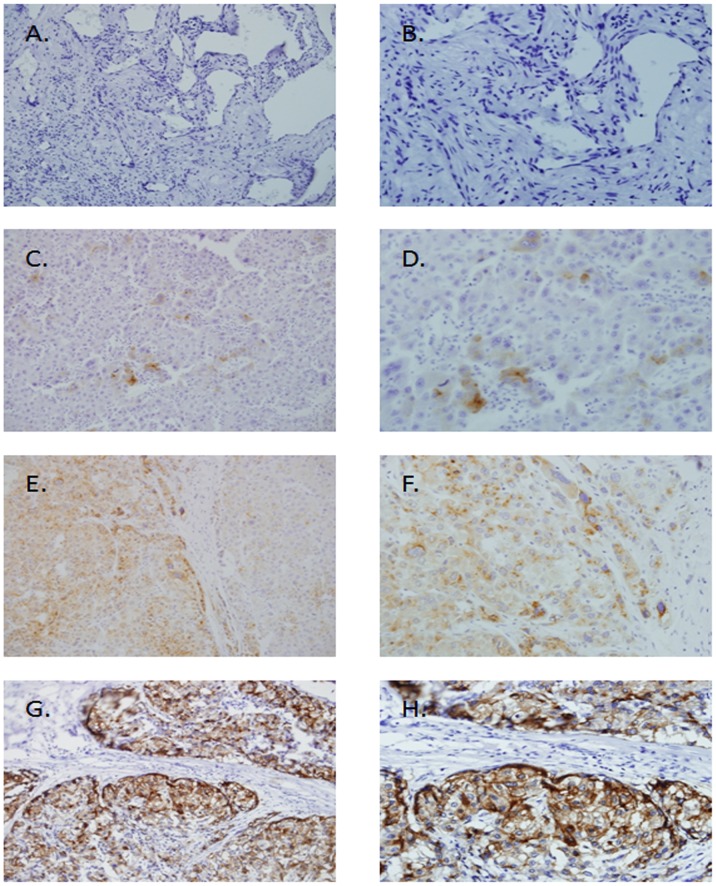
Analysis of CTSL protein in tissues by immunohistochemistry. A and B, CTSL expression is negative in normal liver cells. C and D, CTSL expression is weak in well-differentiated HCC cells. E and F, CTSL expression is moderate in moderately differentiated HCC cells. G and H, CTSL expression is strong in poorly differentiated HCC cells. (A, C, E, G ×200; B, D, F, H ×400).

To verify this observation, we further examined the expression of CTSL protein in 82 paraffin-embedded HCC samples and 16 normal liver (non-cancerous) samples by immunohistochemical analysis. As shown by immunohistochemical analysis, 35 of 82 (42.7%) paraffin-embedded HCC tissues showed weak or negative staining of CTSL protein, while 30 of 82 (36.6%) HCC tissues showed mainly moderate CTSL staining (in the membrane and cytoplasm of cancer cell) and 17 of 82 (20.7%) showed strong staining in tumor cells. Thirteen of the 16 non-cancerous tissues indicated negative staining of CTSL and the rest two non-cancerous tissues showed weak expression (Figure 2).

Additionally, the incidence of CTSL protein expression in well-differentiated carcinoma was significantly lower than that in poor-differentiated tumors, and CTSL expression was significantly related with tumor differentiation (P = 0.007) (Table 1).

**Table 1:** Relationship between CTSL expression and clinicopathologic features of HCC patients.

Clinicopathologic parameters	n	CTSL expression		*P* value
		Positive (%)	Negative (%)	
All cases	82	47(57.3%)	35(42.7%)	
Gender				0.652
Male	61	32(52.5%)	29(47.5%)	
Female	21	15(71.4%)	6(28.6%)	
Age(years)				0.583
<50	58	30(51.7%)	28(48.3%)	
≥50	24	17(70.8%)	7(29.2%)	
Tumor size(cm)^Δ^				0.706
<5	47	26(55.3%)	21(44.7%)	
≥5	35	21(60.0%)	14(40.0%)	
Serum HBsAg				0.342
Positive	66	35(53.0%)	31(47.0%)	
Negative	16	12(75.0%)	4(25.0%)	
Serum AFP(ng/ml)				0.256
<25	30	14(46.7%)	16(53.3%)	
≥25	52	33(63.5%)	19(36.5%)	
Cirrhosis				0.019
Presence	64	40(62.5%)	24(37.5%)	
Absence	18	7(26.7%)	11(72.3%)	
UICC stage				0.026
I+II	59	35(59.3%)	24(40.7%)	
III+IV	23	12(52.2%)	11 (47.8%)	
Metastasis/Recurrence				0.001
Yes	58	40(69.0%)	18(31.0%)	
No	24	7(29.2%)	17(70.8%)	
Edmondson grade				0.007
Low (I/II)	62	32(51.6%)	30(48.4%)	
High (III/IV)	20	15(75.0%)	5(25.0%)	

Δ: The largest dimension of the tumor specimen.

### Correlation of CTSL Expression with Clinicopathological Features and Outcomes

The association between CTSL expression and the clinicopathological outcomes is shown in Table 1. CTSL expression was significantly correlated with liver cirrhosis, stage, Recurrence and tumor differentiation. There was no significant correlation between CTSL expression and age, gender, Tumor size, Serum HBsAg or Serum AFP (Table 1).

### Correlation of CTSL Expression with Overall Survival

**Figure 3:**
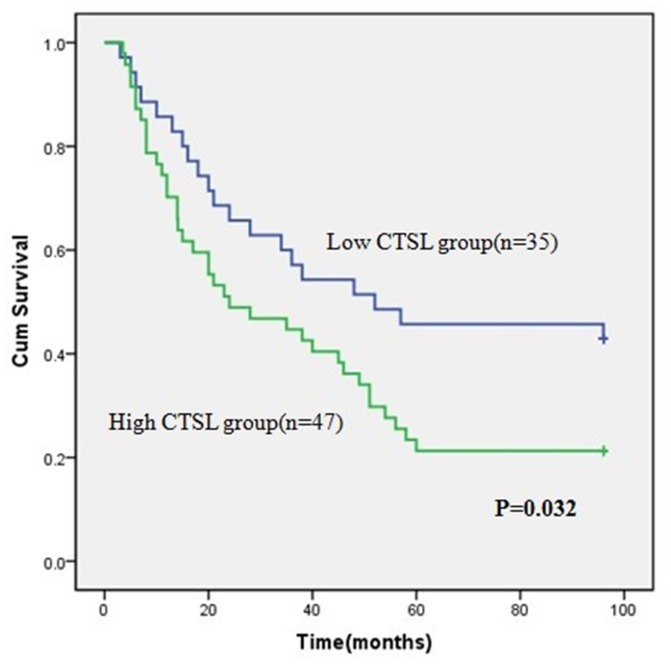
Survival curves for patients with high CTSL expression versus low CTSL-expressing carcinoma. The 5-year overall survival rate was 22.7% in the high CTSL protein expression group (green line), but it was only 41.4% in the low expression group (blue line), P = 0.032.

The median follow-up time for overall survival was 78 months for all patients. The 2-year and 5-year overall rates for all patients were 65.6% and 33.6%, respectively. Among these patients, the overall survival of the patients with low CTSL expression (5-year overall rate, 41.4%) was significantly higher than the high CTSL expression group (5-year overall rate, 22.7%) (P = 0.032, Figure 3). Besides CTSL expression level, age, tumor size, serum AFP, stage, tumor recurrence and tumor differentiation were also significantly correlated with overall survival in univariate analysis (Table 2). Furthermore, overall survival was possibly correlated with liver Cirrhosis (P = 0.093).

**Table 2:** Univariate survival analysis of 82 patients with HCC.

Variable	n	Overall survival		*P* value
		2-year	5-year	
CTSL expression				0.032
Positive	47	50.3%	22.7%	
Negative	35	64.3%	41.4%	
Gender				0.421
Male	61	62.2%	38.6%	
Female	21	64.1%	40.0%	
Age(years)				0.022
<50	58	67.6%	48.5%	
≥50	24	53.2%	30.3%	
Tumor size(cm)^Δ^				0.015
<5	47	65.7%	42.1%	
≥5	35	52.6%	31.7%	
Serum HBsAg				0.103
Positive	66	62.3%	33.9%	
Negative	16	65.1%	37.1%	
Serum AFP(ng/ml)				0.001
<25	30	72.2%	42.3%	
≥25	52	54.1%	21.8%	
Cirrhosis				0.093
Presence	64	63.4%	32.5%	
Absence	18	69.8%	37.2%	
UICC stage				0.001
I+II	59	72.4%	40.1%	
III+IV	23	54.6%	22.5%	
Metastasis/Recurrence				0.000
Yes	58	50.4%	22.1%	
No	24	65.2%	34.6%	
Edmondson grade				0.017
Low (I/II)	62	70.3%	43.1%	
High (III/IV)	20	60.4%	36.8%	

Δ: The largest dimension of the tumor specimen.

The Cox proportional hazards mode was employed to evaluate the effects of the independent factors on overall survival. These factors include CTSL expression, gender, age, tumor size, Serum HBsAg, serum AFP, tumor size, liver cirrhosis, stage, tumor recurrence and tumor differentiation. The results showed that CTSL expression, serum AFP, tumor size, tumor recurrence and stage were recognized as independent prognostic factors of survival (Table 3). Therefore, Multivariate analysis indicated that CTSL protein expression has a significant correlation with poor prognosis of HCC patients as an independent factor.

**Table 3:** Cox regression analysis of patients with HCC.

Variables	Univariate		*P* value
	HR	CI(95%)	
CTSL expression(1 = down, 2 = over)	3.025	2.297∼4.067	0.002
Gender(1 = male, 2 = female)	0.342	0.113∼1.146	0.091
Age(1<50, 2≥50)	0.676	0.412∼1.412	0.352
Serum HBsAg(1 = negative, 2 = positive)	1.466	0.523∼3.736	0.401
Serum AFP(1<25 ng/ml, 2≥25 ng/ml)	2.012	1.152∼3.712	0.017
Tumor size(1<5 cm, 2≥5 cm)	1.756	1.218∼3.321	0.039
Cirrhosis(1 = Absence, 2 = Presence)	1.365	0.643∼2.789	0.302
Metastasis/Recurrence(1 = no, 2 = yes)	3.345	1.730∼6.125	0.000
UICC stage(1 = I+II, 2 = III+IV)	2.141	1.121∼3.568	0.031
Edmondson grade(1 = High (III/IV), 2 = Low (I/II))	0.739	0.361∼1.536	0.563

### CTSL Might Affect the Proliferation and Tumor Progression Ability of MHCC-97H Cells

**Figure 4:**
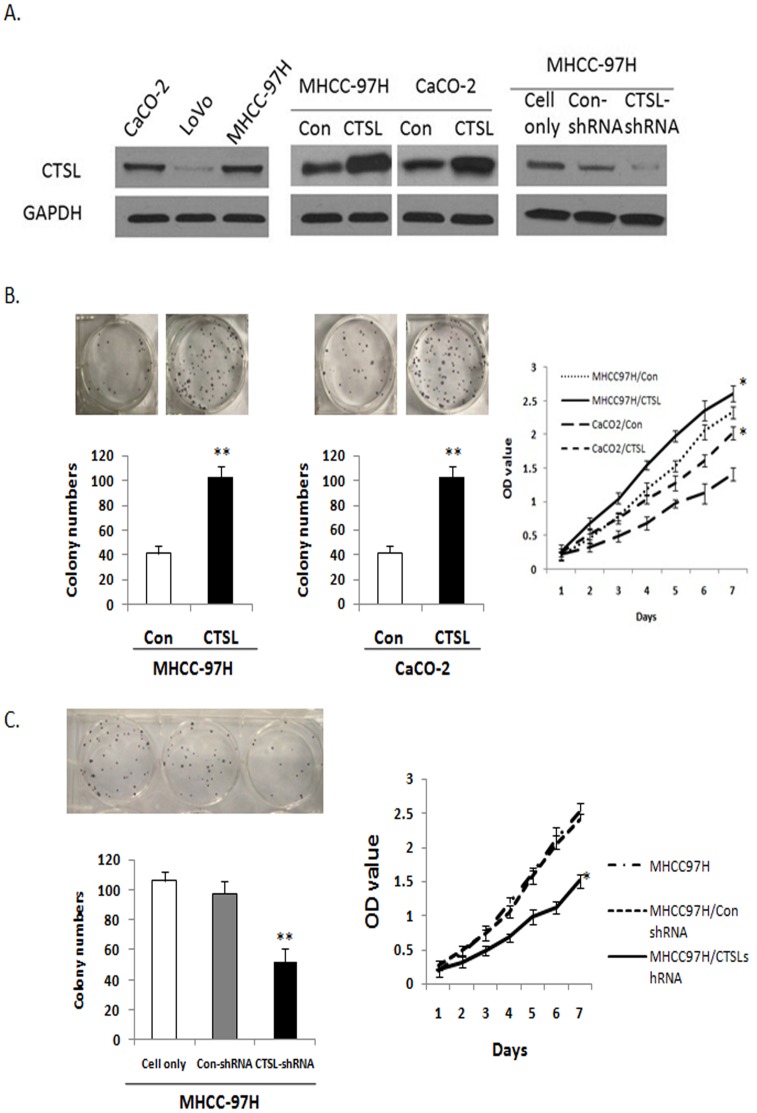
The effect of CTSL on the proliferation and tumor progression ability of MHCC-97H cells. A. Western blotting analysis of CTSL protein expression in HCC cell line (MHCC-97H), colorectal carcinoma cell lines (CaCO2 and LoVo), stably CTSL-expressed MHCC-97H cell line, stably CTSL-expressed CaCO2 cell line, empty vector stable cell lines (MHCC-97H-Con or CaCO2-Con), and MHCC-97H cell line transfected by CTSL-shRNA or Con-shRNA. B. Colony formation assay and MTT assay of MHCC-97H cells and CaCO2 cells with over-expression of CTSL. (Colony formation assay: MHCC-97H-Con (vector) vs MHCC-97H-CTSL, P = 0.0042; CaCO2-Con (vector) vs CaCO2-CTSL, P = 0.0072. MTT: MHCC-97H-Con (vector) vs MHCC-97H-CTSL, P = 0.012; CaCO2-Con (vector) vs CaCO2-CTSL, P = 0.035). C. Colony formation assay and MTT assay of MHCC-97H cells with down-regulated CTSL. (Colony formation assay: MHCC-97H-Con-shRNA vs MHCC-97H-CTSL-shRNA, P = 0.003; MTT: MHCC-97H-Con-shRNA vs MHCC-97H-CTSL-shRNA, P = 0.001. (**P<0.01 as compared to parental groups, *P<0.05 as compared to parental groups).

The protein levels of CTSL of six HCC cell lines were shown in Fig. S1. The data showed that MHCC-97H expressed highest level of CTSL protein and thus was chosen for further study on the biological function of CTSL. In addition, as shown in Figure 4A, the expression level of CTSL was high in MHCC-97H and CaCO2 cells compared to LoVo cells. To further investigate whether CTSL could enhance the proliferation and tumor progression ability of HCC cells (MHCC-97H) and colorectal cancer cell lines (CaCO2), we established stable MHCC-97H cell line and CaCO2 cell line that expressed CTSL (MHCC-97H-CTSL or CaCO2-CTSL) or empty vector (MHCC-97H-Con or CaCO2-Con). Over-expression of CTSL promoted cell proliferation and malignant transforming ability of MHCC-97H cells and CaCO2 cells by colony formation assay and MTT assay (Figure 4B). To further investigate the effect of CTSL in the proliferation and malignant transforming ability of HCC cells (MHCC-97H), we established stable MHCC-97H cell lines with down-regulation of CTSL by shRNA sequences against CTSL (MHCC-97H-CTSL-shRNA). As shown in Figure 4A, the expression level of CTSL was significantly decreased in MHCC-97H-CTSL-shRNA cells compared to control cells (MHCC-97H-Con-shRNA). Knocking-down of CTSL in MHCC-97H cells decreased malignant transforming ability and cell proliferation (Figure 4C), suggesting that over-expression of CTSL might involve in the development of HCC.

### Over-expression of CTSL promoted the Tumor Growth in Nude Mice

**Figure 5:**
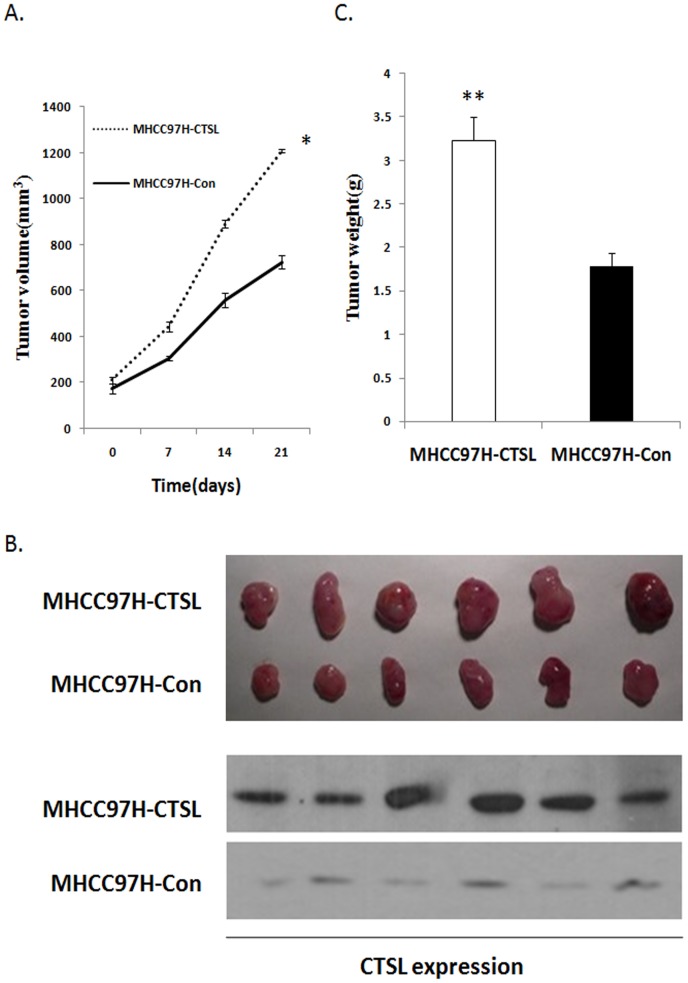
Effect of CTSL knockdown on subcutaneous tumorigencity of MHCC-97H. A. Tumor growth curve of after injection of nude mice with CTSL or control vector expressing MHCC-97H cells. (P<0.001) B. The picture of tumors from nude mice with CTSL or control vector expressing MHCC-97H cells. C. The weight of tumors from nude mice with CTSL or control vector expressing MHCC-97H cells (P = 0.005). (**P<0.01 as compared to control groups, *P<0.05 as compared to control groups).

In vivo experiment was performed to evaluate the effect of CTSL over-expression in nude mice. As shown in Figure 5A and 5B, the growth rate and tumor weight of CTSL tumors were found to be much higher than those with control (MHCC-97H-Con). As shown in Figure.5C, a remarkable increase of tumor size of groups MHCC-97H-CTSL was observed as compared with that of the control group. The result suggested that over-expression of CTSL promoted tumorigenicity of MHCC-97H cells in vivo.

## Discussion

The occurrence and development of HCC are a comprehensive pathologic process involving complex alterations in oncogenes and tumor suppressor genes, which play roles in cell proliferation, cell-cycle control and cell apoptosis through regulation of multiple signal transduction pathways.

The first observed function of CTSL in cancer progression was its ability to promote cancer metastasis [20]. Early experimental studies revealed that the metastatic capability of tumor cells was correlated with CTSL activity. For example, subpopulations of high metastatic potential of murine B16 melanoma cell lines were found to express higher levels of CTSL when compared to their low-metastatic counterparts [21]. The invasive ability of brain tumor cells was markedly reduced by full-length antisense cDNA of CTSL [12]. Moreover, the finding that CTSL contribute to anti-apoptosis is also a well accepted observation experimentally. Enhanced susceptibility of CTSL-deficient A549 lung cells to spontaneous and anti-Fas-induced apoptosis was reported, with a possible mechanism involving altered Cathepsin D processing by CTSL [22]. However, Up to now, little has been known about whether CTSL is involved in HCC progression. Thus, in this study, we tried to investigate the role of CTSL on the development of HCC.

As shown by immunohistochemical analysis in our study, 20.7% paraffin-embedded HCC cancer tissues showed strong membrane and cytoplasm staining of CTSL, 36.6% HCC tissues showed moderate CTSL staining and 42.7% showed negative staining in tumor cells, while the non-cancerous tissues presented mainly negative expression of CTSL, indicating that CTSL might play an important role in the development and progression of HCC. In addition, as determined by immunohistochemistry, the incidence of CTSL protein expression in poor-differentiated carcinoma was significantly higher than that in well-differentiated tumors, suggesting that high level of CTSL expression was related to poor tumor differentiation. Additionally, we have shown that CTSL expression was correlated with liver cirrhosis, stage, Recurrence and tumor differentiation. There was no significant correlation between CTSL expression and age, gender, Tumor size, Serum HBsAg or Serum AFP. Our study suggests that high level of CTSL expression might be positively correlated with worse tumor biological features, such as rapid tumor progression and metastases, and that CTSL plays an important role in the development and progression of HCC. Furthermore, we have shown by multivariate analyses that patients with CTSL protein expression in carcinoma had a poor prognosis than those without CTSL expression, and that serum AFP, tumor size, tumor recurrence and stage and the status of CTSL protein were independent factors influencing overall survival, indicating that CTSL is a powerful prognostic index of survival in HCC. These findings also suggested that clinicopathological features together with detection of CTSL in HCC tissue could be valuable in evaluating prognosis or designing individual therapeutic policy for HCC.

In spite of the potential significance of CTSL in HCC, functional role of CTSL in HCC have not been clearly defined. Demonstration of its oncogenic activity in HCC is still lacking. To understand the functions of CTSL, the endogenous CTSL expression in an HCC cell line (MHCC-97H) was silenced by shRNA. Cell properties of the CTSL-depleted cells were then analyzed and compared with the control cells in various functional assays. The results showed that CTSL knockdown stable clones displayed suppressed cell proliferation ability. Additionally, overexpression of CTSL promoted the aggressive behaviors of MHCC-97H cells. Our study has also provided the first validation about the oncogenic capacity of CTSL expression in vivo. MHCC-97H with high level of CTSL expression displayed increased ability to form tumors in nude mice. All these studies affirmed our findings that CTSL exerts oncogenic effect on MHCC-97H cells.

CTSL expression status, combined with clinicopathological features and other biomarkers of HCC, may be useful to stratify patients for individual treatment, such as those of chemotherapy or TACE(Transcatheter Arterial Chemoembolization). Further investigation in other patient population or group is required to verify these hypotheses. Since the number of the cases in this study was not too big, the relationship between CTSL expression and metastases still requires to be evaluated. A recent study showed that CTSL might promote chemoresistance by their ability to resist various apoptotic stimuli in glioblastoma Cells, however the study was about brain cancer and the case scale was small [8]. Therefore, further studies are needed to clarify the mechanisms by which CTSL is involved in the development and progression of HCC.

Altogether, this study show that the first evidences of the expression and clinical significance of CTSL in HCC, suggesting that CTSL might involve in the development of HCC as a tumor promoter, and thereby may serve as a valuable prognostic marker for HCC patients.

## Supporting Information

Figure S1 **Expression of CTSL in six human HCC cell lines.** CTSL protein expression levels in MHCC-97H, MHCC-97L, Huh-7, HepG2, SMMC-7721 and Bel-7404 cell lines were determined by Western blot. MHCC-97H showed the highest level of CTSL as compared to the rest cell lines.
(TIF)
