# Supplementary figures and images for: Increased Expression of Cathepsin L: A Novel Independent Prognostic Marker of Worse Outcome in Hepatocellular Carcinoma Patients
Source: PLoS One. 2014 Nov 10;9(11):e112136. doi: 10.1371/journal.pone.0112136 (PMC4226473; doi:10.1371/journal.pone.0112136)

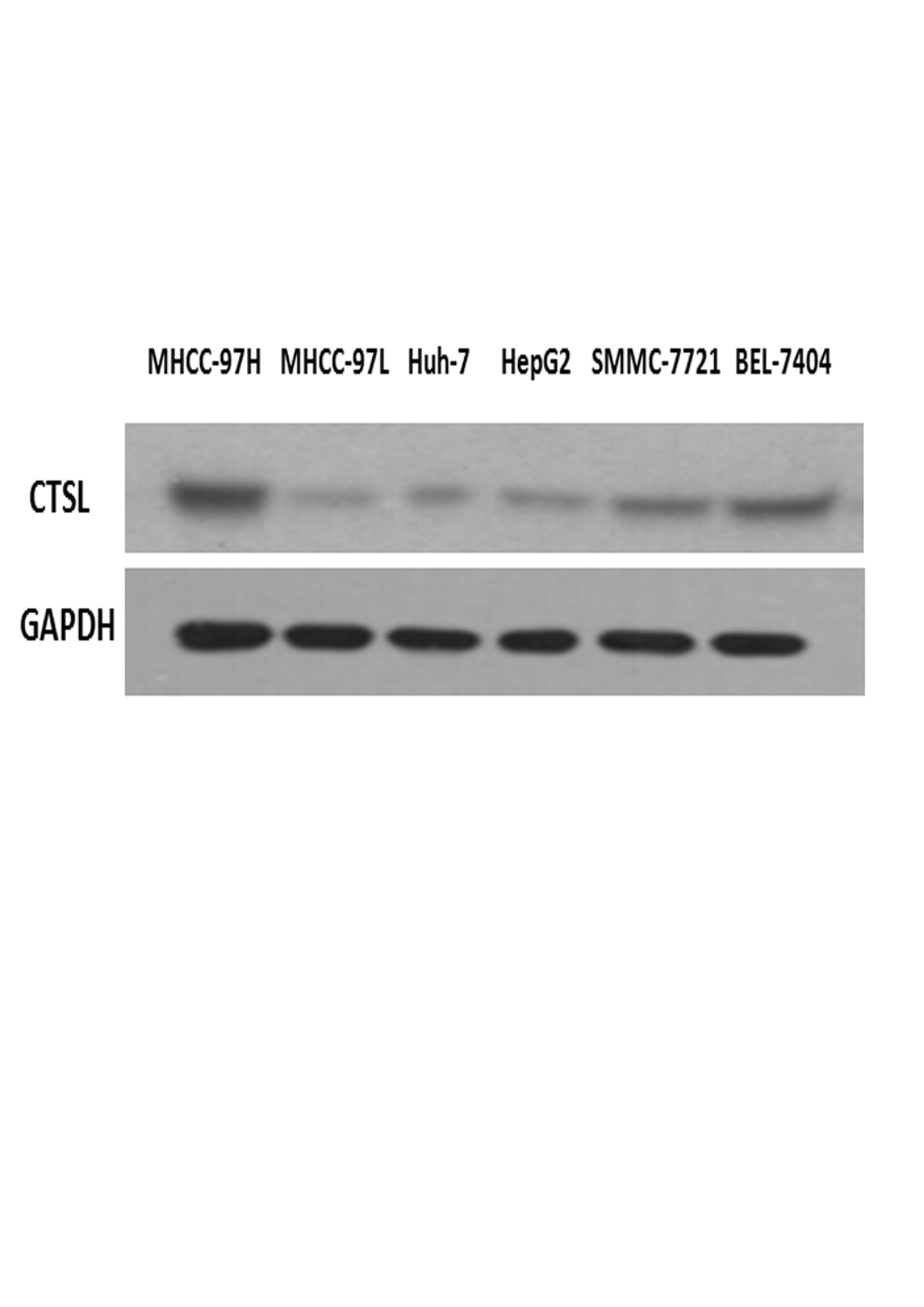

Supplement: Figure S1 — Expression of CTSL in six human HCC cell lines. CTSL protein expression levels in MHCC-97H, MHCC-97L, Huh-7, HepG2, SMMC-7721 and Bel-7404 cell lines were determined by Western blot. MHCC-97H showed the highest level of CTSL as compared to the rest cell lines. (TIF) [file pone.0112136.s001.tif]
